# The Prevalence, Risk Factors, and Clinical Outcomes of Vitamin C Deficiency in Adult Hospitalised Patients: A Retrospective Observational Study

**DOI:** 10.3390/nu17071131

**Published:** 2025-03-25

**Authors:** Janet Golder, Judith Bauer, Lisa A. Barker, Christopher Lemoh, Simone Gibson, Zoe E. Davidson

**Affiliations:** 1 Department of Nutrition, Dietetics and Food, School of Clinical Sciences, Faculty of Medicine, Nursing and Health Sciences, Monash University, Level 1, 264 Ferntree Gully Road, Notting Hill 3168, VIC, Australia; janet.golder@monash.edu (J.G.); judy.bauer@monash.edu (J.B.); lisa.barker@monash.edu (L.A.B.); 2 Allied Health, Monash Health, 400 Warrigal Rd., Cheltenham 3192, VIC, Australia; 3 Department of Medicine, School of Clinical Sciences at Monash Health, Faculty of Medicine, Nursing and Health Sciences, Monash University, 246 Clayton Rd., Clayton 3168, VIC, Australia; christopher.lemoh@monash.edu; 4 Department of Medicine at Western Health, Melbourne Medical School, The University of Melbourne, WCHRE Building, Level 3, 176 Furlong Road, St Albans 3021, VIC, Australia; 5 School of Clinical Sciences, Faculty of Medicine, Nursing and Health Sciences, Monash University, 246 Clayton Rd., Clayton 3168, VIC, Australia; simone.gibson@monash.edu

**Keywords:** ascorbic acid, vitamin C deficiency, scurvy, prevalence, malnutrition

## Abstract

**Background/Objectives**: Assessment of vitamin C status rarely occurs in hospital patients within high-income countries on the assumption that vitamin C deficiency (VCD) is rare, and evidence on prevalence, risk factors, and clinical outcomes of VCD is limited. This study aimed to describe the prevalence of VCD, characteristics of patients with VCD, and identify risk factors and clinical outcomes associated with VCD status in adult hospitalised patients. **Methods**: This retrospective observational study included adult inpatients from five metropolitan hospitals within a single public health service in Australia which provides tertiary, acute, and sub-acute care, over a 3.5-year period. Non-fasting vitamin C levels were examined for the prevalence of VCD, defined as <11.4 µmol/L. Multivariate regression models were used to identify risk factors and clinical outcomes associated with VCD. **Results**: The prevalence of VCD was 22.9% (n = 1791), comprising 23.2% (n = 1717) and 16.2% (n = 74) within acute and sub-acute settings, respectively. VCD prevalence was high in acute setting subgroups including patients with malnutrition (30%, n = 611) and patients admitted to ICU during hospitalisation (37.3%, n = 327). Malnutrition (OR 1.50, 95% CI 1.19–1.91, *p* < 0.001) and male gender (OR 1.47, 95% CI 1.17–1.86, *p* = 0.001) were associated with VCD. VCD was not associated with clinical outcomes including in-hospital death, hospital or intensive care unit LOS, or hospital-acquired complications. **Conclusions**: VCD exists within adult hospital patients in high-income countries, and early, targeted detection of VCD in this setting is warranted. Further research is needed to explore the impact of VCD on hospital clinical outcomes.

## 1. Introduction

Vitamin C (or ascorbic acid) has many important functions during acute illness, including the synthesis of hormones including vasopressin and noradrenaline [1] and neurotransmitters such as dopamine, norepinephrine and serotonin [2]. Vitamin C also supports iron absorption, energy production via carnitine synthesis [3,4], formation of bile acids in the gastrointestinal tract [4], and collagen synthesis [5]. It acts as a potent antioxidant during acute infection and inflammation [6] and in turn, supports the regeneration of other antioxidants such as α-tocopherol [7]. Recent research also suggests a role for vitamin C in cellular stress-signalling and epigenetics [7]. Despite its physiological importance, assessment of vitamin C status rarely occurs in hospital patients within high-income countries. Although VCD is present in adults within the community setting, reported as 1.4% (n = 22,474) in England [8] and 7% (n = 13,824) in the United States [9], there is an assumption that deficiency status is unlikely in hospital patients, given the widespread availability of vitamin C-rich foods and the high consumption of vitamin C supplements [3]. Vitamin C deficiency (VCD) is defined as a plasma vitamin C level < 11.4 µmol/L based upon the WHO reference standards [10]. A recent scoping review reported that the prevalence of VCD in adult hospitalised patients in high-income countries (defined by the Organization for Economic Co-operation) [11] was 27.7% (n = 2494) [12] according to this diagnostic criterion. High prevalence of VCD was observed within inpatient subgroups, reported as 38% (n = 24) [1] and 48.9% (n = 111) [13] in critically ill patients with septic shock, 50.8% (n = 131) in patients with diabetic foot ulcers [14], 45% (n = 30) in oncology patients with a poor nutrition status [15], and 41.6% (n = 149) in general medical patients [3]. Although compelling, the prevalent studies within this review were conducted in small sample populations, or were targeted to specific hospital subgroups selected based on their higher risk of deficiency related to diagnosis or admission setting. No evidence exists regarding the prevalence of VCD in a large study group within an adult patient population selected from an entire hospital setting, which reflects a pragmatic and operational perspective of this observation.

Similarly, there is limited evidence about the risk factors which can predict VCD status specific to adult hospitalised patients. Demographic and clinical characteristics of hospital patients such as age [16], male gender [17,18], smoking [16,19], and increased inflammatory markers [18] have shown an association with VCD, yet this association has not been consistently reported, or has been in single studies with small sample sizes and analysed via univariate models only. A relationship between VCD and malnutrition has previously been demonstrated [12], yet studies used unvalidated variables to diagnose PEM. A recent study [20] observed that the prevalence of VCD increased with the severity of protein–energy malnutrition (PEM) according to subjective global assessment (SGA) [21] and global leadership initiative on malnutrition (GLIM) [22] malnutrition diagnosis methods, yet an association between these variables and VCD was not established, limited by the lack of well-nourished controls [20]. To date, only concomitant excess alcohol and tobacco (*p* = 0.0003) and being retired (*p* = 0.015) [18] have been identified as independent risk factors for VCD via multivariate analysis, within a single small study (n = 184) conducted in a specific general medical cohort. A similar paucity of evidence exists for establishing the clinical impact of VCD on patient outcomes or hospital performance metrics. VCD is reported to be a predictor for increased risk of moderate-severe frailty [23] and cognitive impairment (n = 160) [19], and yet the association between VCD status using the WHO diagnostic criteria [10] and important hospital performance measures such as length of stay (LOS), in-hospital death, and reportable hospital-acquired complications has not yet been examined.

The purpose of this large retrospective observational study is to describe the prevalence of VCD in a typical tertiary hospital cohort of adult inpatients in a high-income country; describe the characteristics of patients with VCD; explore potential risk factors which could predict VCD status; and explore clinical outcomes associated with deficiency status.

## 2. Materials and Methods

### 2.1. Study Design and Patients

**Figure 1:**
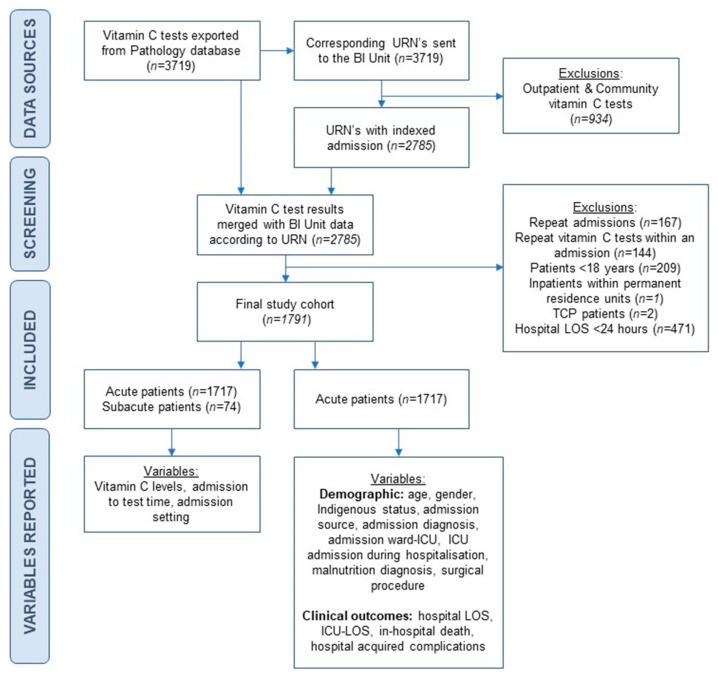
Flowchart describing data sources, patient selection and variables reported in the final study cohort and acute subgroup. BI Unit: Business Intelligence Unit; ICU: intensive care unit; LOS: length of stay; TCP: transition care program; URN: unique registration number.

This was a retrospective observational study of patients admitted to five metropolitan hospital sites within a single health service in Melbourne, Victoria, Australia, providing tertiary, acute, and sub-acute care. Quality assurance ethics approval for the study as a clinical audit was provided by Monash Health (RES-23-0000-001Q) and Monash University (project 37318) human research ethics committees; individual patient consent was not required. Refer to Figure 1 for a flowchart describing data sources, patient selection, and variables reported.

Overall methodology was pragmatically focused, utilising existing routine clinical data available for health service reporting. The study period was from 1 January 2017 to 30 June 2020. Only patients who had vitamin C tested within this timeframe were included; these patients were identified through the health service Pathology Department database. For those patients who had an indexed hospital admission, admission data which corresponded to a vitamin C test date was then obtained from the health service Business Intelligence Unit. The Pathology Department and Business Intelligence Unit datasets were merged, and exclusions were applied.

Each patient was represented only once during the study period; where a patient had multiple admissions during the study period, only data from the first (earliest) admission where vitamin C was tested was included. If a patient had multiple vitamin C tests during that first admission, only the first (earliest) plasma vitamin C test result was included in data analysis. The inclusion criteria were adult inpatients (≥18 years) within acute or sub-acute hospital wards, who had plasma vitamin C tested during admission. The following exclusions were applied: paediatrics (<18 years); adults residing within the community setting including outpatients and transition care programme patients; adults living within health service residential mental health units and residential aged care facilities; adult inpatients who had a hospital LOS < 24 h; and those who were admitted directly to a palliative care unit. Via this methodology, aetiological components of PEM (including dietary intake and inflammation) [22] could not be identified, and inpatients taking vitamin C supplementation could not be excluded from the dataset. Patients from the one sub-acute site (n = 74) were included within the prevalence analysis only. The remaining analyses were conducted within the remaining four acute sites.

Figure 1 outlines the variables reported within the final merged dataset. Patients’ primary diagnosis was reported according to Australian government Diagnostic Related Group categories [24]. At this health service, PEM may be diagnosed at admission or throughout the hospital admission by a doctor using the ICD-10 criteria [25] or by dietitians who are trained in the use of the subjective global assessment tool (SGA) [21]. Nursing-led nutrition risk screening routinely occurs on admission for all inpatients. Patients who are identified as at high risk of malnutrition according to nutrition screening tools are referred to the dietitian, who then complete an initial nutrition assessment as part of routine clinical practice which includes completion of the SGA. Patients coded as malnourished in the dataset includes those diagnosed with unspecified severe PEM (E43.0), moderate PEM (E44.0), mild PEM (E44.1), and unspecified PEM (E46) according to ICD-10-AM (Australian modification) classification [26], diagnosed as either a primary code or a complication code when serial nutrition assessment determined that PEM developed during hospitalisation. Patients without a PEM diagnosis included those assessed as well-nourished, and those who were not assessed; this difference was not distinguishable in the dataset. Surgical procedure during admission included all procedure types (elective or emergency surgery). Admission ward-ICU refers to patients admitted directly into intensive care unit (ICU).

Clinical outcomes included hospital LOS, ICU-LOS, in-hospital death, and hospital-acquired complications reported according to the Australian Commission on Safety and Quality in Healthcare [27], including delirium, gastrointestinal bleeding, high-impact infections, pneumonia, and respiratory failure [27]. Clinical signs and symptoms of scurvy were unable to be collected in the absence of medical file review.

No data were missing from the dataset.

### 2.2. Biochemical Analysis

Vitamin C analysis was conducted by the health service Pathology Department. Standard protocol requires the collection of blood samples for vitamin C analysis in EDTA tubes containing didehydrothreonine (an antioxidant which prevents oxidation of ascorbic acid to dehydroascorbic acid), and for them to be transported on ice immediately to the laboratory. Upon arrival, a protein precipitating agent containing metaphosphoric acid and didehydrothreonine was added to the sample and mixed thoroughly. After centrifugation, the supernatant was stored at −20 °C, until analysis by high-performance liquid chromatography (HPLC). The limit of detection for plasma vitamin C is 5 µmol/L; vitamin C levels reported as <5 µmol/L were recoded to a value of 4.9 µmol/L for statistical analysis. In accordance with local protocols, patients were not required to fast for vitamin C testing.

VCD is defined as a plasma vitamin C < 11.4 µmol/L using the internationally recognised definition [10]. Normal vitamin C status is defined locally as ≥23 µmol/L. Therefore, for this study vitamin C depletion status was defined as 11.4–22.9 µmol/L.

### 2.3. Statistical Analysis

All statistical analysis was conducted in SPSS Statistics (IBM, New York, NY, USA, version 28). The prevalence of VCD was determined as a proportion calculation of patients who were VCD within the specified cohorts who had vitamin C tested. VCD was retained as a dichotomous categorical dependent variable for analysis, as this reflects diagnostic criteria which underpins clinical decision making in the hospital environment. Categorical variables are presented as frequency and percent (n, %). Continuous variables were assessed for normality using the Shapiro–Wilk test; as all continuous variables showed skewed distributions, and data are presented as the median and interquartile range. Any outliers present within continuous variables were retained in the dataset as none met valid criteria for removal. All PEM codes assigned as an initial or complication diagnosis were grouped together for descriptive statistics and multivariate models to explore the association between malnutrition and VCD irrespective of assessment period. ICU-LOS was analysed within patients who were admitted into ICU. Hospital LOS and ICU-LOS were adjusted for in-hospital death. Characteristics of patients with VCD were compared to those without VCD. Chi-square was used to test the association between VCD and categorical variables for potential use in prediction models. The Mann–Whitney U test was used to test for associations between VCD and continuous variables of interest.

Regression analysis was used to explore risk factors for VCD, and variables for inclusion were based on clinical plausibility, research evidence, and a *p*-value of <0.10 via Chi squared or Mann–Whitney U tests. Variables showing significant association (set at *p*-value *<* 0.10) via univariate generalised linear mixed model (GLMM) logistic regression analysis in all models underwent collinearity diagnostics using Pearson’s correlation (with collinearity set at an r-vale of <0.8) followed by linear regression for assessment of variance inflation factor set at <0.3. Only variables meeting statistical significance and collinearity diagnostics underwent multivariate GLMM logistic regression analysis. All GLMM logistic regression models used a random intercept to adjust for hospital level variance, given that data were collected from four different acute hospital sites. The sample size for models used the formula n = 100 + 50i where i refers to number of independent variables [28]. Results for each predictor variable are presented as odds ratio (OR), 95% confidence interval (CI), and a *p*-value set at <0.05 for statistical significance in final models.

Similar regression analysis was used to identify clinical outcomes associated with VCD in the presence of other plausible predictor variables available in the dataset. Variables underwent univariate GLMM logistic regression for testing an association with dichotomous categorical dependent variables, or univariate GLMM negative binomial regression for hospital and ICU-LOS, presented as count outcome variables [29]. The univariate and multivariate analysis was conducted for all clinical outcomes as described above, retaining the same random intercept. A statistician from the university was consulted for the statistical analysis strategy.

## 3. Results

### 3.1. Study Population

A total of 1791 adult patients met inclusion criteria for prevalence analysis in this study. This included patients admitted into acute care wards (from tertiary and acute hospitals) (n = 1717) and sub-acute wards (n = 74) across the five health service hospital sites. Only patients admitted into acute care wards (n = 1717) were included in the analysis of risk factors and clinical outcomes associated with VCD status.

The overall median vitamin C level within acute patients was 27.1 (12.1, 49.9) µmol/L, and the median time from admission to vitamin C test was 4 (2, 8) days. The median age at admission was 65 years old, gender was evenly dispersed across males and females, and most patients were admitted from home and discharged back to their private residence. Patients were admitted under 35 different medical units, predominantly under general medicine (48.9%). The most common primary diagnosis groups were digestive diseases and respiratory diseases. PEM was diagnosed in 611 patients (35.6%); this includes malnutrition reported upon initial assessment (n = 511) and as a hospital-acquired complication (n = 100). A total of 731 patients (42.6%) underwent a surgical procedure during admission (Table 1).

### 3.2. Prevalence of VCD

Within the final study cohort (n = 1791), 22.9% of patients had VCD, 29.3% had depleted vitamin C status, and 47.8% had normal vitamin C status. Prevalence of VCD in sub-acute wards was 16.2% (n = 74). Within patients admitted into acute care wards (n = 1717), 23.2% had VCD, 20.5% had depleted vitamin C status, and 56.3% had normal vitamin C status. Of the patients in the acute care wards, 135 had vitamin C levels below the limit of detection via HPLC analysis (<5 µmol/L); this corresponds to 33.8% of the patients with VCD (n = 399), or 8% of the acute cohort (n = 1717).

The prevalence of VCD was investigated within specific acute subgroups and reported as 20.7% (n = 839) in general medicine patients, 30% (n = 611) in patients with a PEM diagnosis, and 37.3% (n = 327) in patients who had an ICU admission during hospitalisation.

### 3.3. Characteristics of Patients with VCD Within Acute Care Wards

Analysis revealed an association between VCD and male gender, malnutrition diagnosis, specific primary diagnosis groups (respiratory disease, digestive disease, or skin/subcutaneous tissue/breast disease), surgical procedure during admission, and admission to hospital from a residential aged care facility or hospital transfer (Table 1). When adjusted for in-hospital death, patients with VCD had a 2-day longer hospital LOS (*p* = 0.008, n = 1635) and a 1-day longer ICU-LOS (*p* = 0.026, n = 282). VCD was also associated with hospital-acquired complications including gastrointestinal bleeding, delirium, high-impact infections, pneumonia, and respiratory failure. VCD status was not significantly associated with in-hospital death (Table 2).

### 3.4. Risk Factors Associated with VCD Status

GLMM logistic regression was performed to explore the association between demographic and clinical variables and VCD (Table 3). The multivariate GLMM logistic regression analysis suggested that PEM (OR 1.50, 95% CI 1.19–1.91, *p* < 0.001), male gender (OR 1.47, 95% CI 1.17–1.86, *p =* 0.001), digestive disease (OR 1.36, 95% CI 1.02–1.82, *p* = 0.037), and surgical procedure (OR 1.31, 95% CI 1.02–1.68, *p =* 0.037) were associated with VCD in adult hospitalised patients.

### 3.5. VCD Status Associated with Hospital Clinical Outcomes

GLMM logistic regression was performed to explore the association between VCD (and other clinically relevant predictors), and a range of hospital clinical outcomes.

VCD was not associated with gastrointestinal bleeding complications or adjusted hospital LOS via univariate GLMM. Within multivariate GLMM, in the presence of relevant clinical variables available within the dataset, VCD status was also not associated with delirium, pneumonia, respiratory failure, or adjusted ICU-LOS. When included in a multivariate GLMM logistic regression model with malnutrition as a univariate predictor, VCD was associated with high-impact infection complications (OR 1.72, 95% CI 1.03–2.87, *p* = 0.038) (Supplement Table S1).

## 4. Discussion

Our study revealed that VCD exists in adult hospitalised patients, a finding which adds to the growing level of evidence which challenges the assumption of VCD rarity in adult patients hospitalised in high-income countries. Protein–energy malnutrition (PEM) and male gender were associated with VCD status. Using GLMM, VCD status was not associated with hospital- or ICU-LOS, in-hospital death, or hospital-acquired complications. These findings represent the largest study to date to explore VCD within an inpatient setting, across a typical, hospital wide patient cohort, and is the first study to observe an association between VCD and PEM using validated malnutrition diagnosis methods.

In this study, the overall prevalence of VCD was 22.9% (n = 1791). These data are similar to findings in a recent scoping review [12] which reported the prevalence of VCD in adult hospitalised patients as 27.7% (n = 2494). In comparison to community-dwelling adults where the prevalence of VCD has been reported to range from 1.4% (n = 22,474) [8] in the UK to 7% (n = 13,824) in the USA [9], the prevalence of VCD is higher in hospitalised patients. It is thought that vitamin C status is influenced by multiple factors associated with acute illness and hospitalisation itself which impact vitamin C intake [30,31,32], absorption [33,34,35,36], losses [37], or requirements [1], and contribute to the decline in vitamin C status observed prior to and during admission. Challenges exist in comparing prevalence data when methods vary between studies. Yet despite the patient population in this study having vitamin C status tested on the basis of clinical concern for deficiency, the prevalence of VCD in this study is similar to findings reported in prospective VCD prevalence studies conducted in hospital patients within other high-income countries [17,19,23,38,39,40]. Furthermore, high prevalence of VCD was observed in patients who had an ICU admission during hospitalisation, and this too is consistent with prevalence data in critical care settings reported elsewhere [1,13]. Low vitamin C status in critically ill patients may be a result of rapid vitamin C consumption in response to severe infection and inflammation [1], and recently released clinical guidelines suggest that these patients require additional vitamin C well-beyond the recommended dietary intake in order to achieve plasma concentrations in the high normal range [41].

In contrast to our findings, several other studies reported a higher prevalence of VCD in adult inpatients. Across acute settings including intensive care, general medical, medical oncology, medical and surgical, and vascular surgery, the prevalence of VCD ranges from 30% to 50.8% [1,3,14,15,16,42,43,44]. The lower prevalence of VCD in this study may be a result of heterogeneity in the patient population; namely, the inclusion of patients from all admission units, rather than limiting to subgroups at higher risk of VCD based on diagnosis or hospital setting. The inclusion of sub-acute wards in overall prevalence data, a hospital setting where lower VCD prevalence rates similar to these findings have been reported elsewhere [40,45], may also contribute. The prevalence of VCD may also be under-estimated in this study, associated with non-fasting blood samples for analysis given that plasma vitamin C follows a steep sigmoidal relationship with recent dietary intake of vitamin C [46], and the inability to exclude patients taking vitamin C supplementation from the dataset. An interesting observation also from the prevalence data in this study was the high proportion of patients with VCD who had undetectable vitamin C status (33.8%, n = 399). The prevalence of undetectable vitamin C status is rarely reported [14,47], yet these data are important as clinical signs and symptoms of scurvy are far more likely to be present in patients with undetectable levels of vitamin C [48], particularly in patients who are severely malnourished [20].

Several demographic and clinical variables were associated with VCD, a finding that may support early identification of patients at risk of deficiency status during admission. High prevalence of VCD was identified in malnourished patients in this study (30%, n *=* 611). This is consistent with prevalence findings reported in other malnourished cohorts in high-income countries, ranging from 39% (n = 54) [42] to 45% (n = 30) [15]. In this study, a relationship between PEM and VCD was demonstrated via multivariate analysis, using validated malnutrition assessment methods commonly used in clinical practice. It is well established that multiple forms of malnutrition, such as PEM and micronutrient deficiencies, can coexist simultaneously in hospital patients [49] given the similar aetiology between micro- and macronutrient deficiency states; specifically, reduced food intake and high disease burden/inflammation [22]. Whilst this was not unexpected, the importance of confirming the relationship between PEM and VCD relates to implications for clinical practice. Use of validated nutrition assessment methods to diagnose adult hospital patients with PEM is the recommendation of peak international clinical nutrition and research experts [50] and has been adopted widely in Australia [51]. As challenges exist with the accuracy of food intake methods [52] and the relationship between VCD and inflammatory markers has been inconsistently reported [12], PEM diagnosed as part of routine clinical practice may offer a pragmatic ‘proxy’ predictor of VCD in the hospital setting. There is the potential that patients at risk of VCD could be identified earlier during hospitalisation through PEM screening and assessment processes in hospitals where these practices routinely occur as part of admission processes [51].

Males have previously been reported to be at higher risk of developing VCD than females in the community setting [9], yet there is inconsistency with this relationship reported in hospitalised patients [17,18,19]. This study identified an association between male gender and VCD in adult hospitalised patients. Poorer vitamin C status in males may be related to lower fruit and vegetable intake [53], lower vitamin C supplement use [9], and greater oxidative stress associated with a higher consumption of alcohol and tobacco [54,55]. Yet as males generally have a higher body weight than females, lower vitamin C levels may in part be related to volumetric dilution, given that weight affects the vitamin C dose–concentration relationship [56]. Nonetheless, this finding suggests that male patients may benefit from more targeted assessment of dietary vitamin C intake and clinical signs and symptoms of scurvy [10] to support earlier identification of VCD during admission. Consistent with other studies [17,19], no association was demonstrated between age and VCD. This observation may be explained by high fruit and vegetable intake [57], low smoking rates [54], low alcohol consumption [55], and high vitamin C supplement use in older patients [9].

No clinical outcomes were associated with VCD in this study. Low vitamin C levels (<28 µmol/L) have previously been associated with a 2-day longer hospital LOS (*p* = 0.02) and a 4-fold increased odds of hospital LOS > 5 days (aOR 4.2, 95% CI 1.56, 11.58) in general medical inpatients [3]. In this study, the authors propose that patients with normal vitamin C stores may be able to better withstand oxidative stress associated with acute illness, potentially resulting in shorter LOS [3]. However, despite patients within this study also reporting an (adjusted) 2-day longer hospital LOS (*p* = 0.008), the association with VCD was not significant in multivariate analysis. This may be related to differences in study design, patient populations, inclusion criteria, higher vitamin C cut-off for analysis, and higher associated prevalence of VCD in the earlier study, or it may be that a true non-association exists. Other studies investigating the relationship between VCD and gastrointestinal bleeding have shown mixed results. Similar to our findings, no association between VCD and gastrointestinal bleeding was reported in general medical patients [3], yet in a cohort of patients admitted with upper gastrointestinal bleeding [58], multivariate analysis showed a significant association between VCD and the adverse event variceal bleeding. However, as both of these studies used a higher cut-off for VCD status within clinical outcome analysis (<28 µmol/L and <23 µmol/L, respectively), the comparison of findings is limited. Furthermore, although the odds of developing a high-impact infection were 1.7-fold higher in patients with VCD, we advise caution in the interpretation of these findings. A bidirectional relationship may exist between VCD and high-impact infection, evident in the case of acute respiratory infections which are a common source of sepsis [38,59]. Carr et al. [38] indicates that patients with pneumonia may have higher consumption of vitamin C during acute respiratory tract infections, yet patients with pneumonia may also have lower baseline levels of vitamin C, potentially increasing susceptibility to infection. Given the retrospective study design, it is also possible that VCD may be a confounding variable for predictors not included in this analysis, as indicated by high Akaike corrected criterion within regression models.

Strengths of this study include the large sample size, heterogeneity of the study cohort which represents a typical hospital-wide patient population, use of validated methods to assess PEM, and exclusive use of health service data which limits reporting error compared to chart review. However, several limitations exist. This is a single health service study which may limit the generalisability of results to other inpatient settings. As discussed above, the prevalence data does not represent the ‘true’ prevalence of VCD within this health service due to limitations of the retrospective design, particularly potential selection bias whereby patients were tested because of a clinical concern about vitamin C status. Pragmatic use of non-fasting samples for plasma vitamin C analysis and an inability to exclude patients taking supplemental vitamin C limits the comparison of prevalence data to other studies with different inclusion criteria. Also, an inability to identify well-nourished patients, or include vitamin C intake and inflammatory markers in VCD prediction analysis may impact the relationship between malnutrition and VCD. Despite the large number of patients with undetectable vitamin C levels, the inability to collect latent and manifest scurvy signs and symptoms limits the ability to explore both the prevalence of scurvy features and the influence on health service performance metrics. Finally, conducting statistical models based only on routinely reported health service data may have excluded important predictor variables.

## 5. Conclusions

This large retrospective observational study has identified that VCD exists in adult hospital patients within a high-income country, and that specific patient subgroups such as those admitted to ICU during hospitalisation and patients with PEM may experience higher rates of deficiency status. This study highlights the need for health professionals to consider patients at risk of VCD during patient clinical assessment practices at admission in order to support earlier detection. Future research perspectives should include prospectively designed studies, such as point prevalence analysis, in order to examine the ‘true’ prevalence of VCD in a typical acute hospital cohort. In targeted patient cohorts, observational studies which explore clinical outcomes of VCD, including the influence of latent and manifest scurvy features, are also warranted.

## Figures and Tables

**Table 1:** Baseline characteristics of adult patients admitted into acute care wards (n = 1717) characterised by VCD status (defined as plasma vitamin C level < 11.4 µmol/L).

Patient Demographics	Total Cohort (n = 1717)	VCD Cohort (n = 399)	Not VCD Cohort (n = 1318)
Age at admission in years (MD, IQR)	65 (49, 78)	63 (50, 75) *	66 (48, 79)
Gender:			
Male (n, %)	860 (50.1)	230 (57.6) ^††^	630 (47.8)
Female (n, %)	857 (49.9)	169 (42.4)	688 (52.2)
Indigenous status:			
Not Aboriginal or Torres Strait Islander (n, %)	1682 (98)	386 (96.7) ^†^	1296 (98.3)
Aboriginal and/or Torres Strait Islander (n, %)	22 (1.3)	6 (1.5)	16 (1.2)
Question not able to be asked (n, %)	13 (0.7)	7 (1.8)	6 (0.5)
Admitted from:			
Home (n, %)	1333 (77.6)	304 (76.2)	1029 (78.1)
Residential aged care facility (n, %)	58 (3.4)	5 (1.25) ^†^	53 (4)
Hospital transfer (n, %)	300 (17.5)	85 (21.3) ^†^	215 (16.3)
Other (n, %)	26 (1.5)	5 (1.25)	21 (1.6)
Admission ward-ICU (n, %)	95 (5.5)	30 (7.5) ^†^	65 (4.9)
ICU admission during hospitalisation (n, %)	327 (19)	122 (30.6) ^††^	205 (15.6)
Major diagnostic category:			
Respiratory (n, %)	201 (11.7)	28 (7) ^††^	173 (13.1)
Circulatory (n, %)	136 (7.9)	25 (6.3)	111 (8.4)
Digestive (n, %)	360 (21)	115 (28.8) ^††^	245 (18.7)
Hepatobiliary and pancreas (n, %)	84 (4.9)	22 (5.5)	62 (4.7)
Musculoskeletal and connective tissue (n, %)	157 (9.1)	34 (8.5)	123 (9.3)
Skin, subcutaneous tissue and breast (n, %)	132 (7.7)	21(5.3) ^†^	111 (8.4)
Endocrine, nutrition and metabolic	89 (5.2)	24 (6)	65 (4.9)
Infectious and parasitic diseases (n, %)	104 (6.1)	26 (6.5)	78 (5.9)
Other (n, %)	454 (26.4)	104 (26.1)	350 (26.6)
Malnutrition diagnosis (n, %)	611 (35.6)	183 (45.9) ^††^	428 (32.5)
Surgical procedure (n, %)	731 (42.6)	209 (52.4) ^††^	522 (39.6)

VCD, vitamin C deficiency; MD, median; IQR, interquartile range; ICU, intensive care unit. Significantly different from the not VCD cohort; * (*p* < 0.10) by Mann–Whitney U test, ^†^ (*p* < 0.05) by Chi square, ^††^ (*p* < 0.001) by Chi square.

**Table 2:** Clinical outcomes associated with VCD (defined as plasma vitamin C level < 11.4 µmol/L) in adult patients admitted into acute care wards (n = 1717).

Clinical Outcomes	Total Cohort (n = 1717)	VCD Cohort (n = 399)	Not VCD Cohort (n = 1318)
LOS (hospital) in days (MD, IQR)	12.8 (6.2, 27.1)	14.4 (7.21, 31.2) *	12.4 (5.9, 25.9)
Adjusted for in-hospital death (n = 1635)	12.6 (6.1, 26.8)	14.4 (7.2, 31.3) *	12.0 (5.9, 25.0)
ICU-LOS (admission ward-ICU) in days, n *=* 95 (MD, IQR)	4.1 (1.8, 8.1)	4.7 (2.6, 7.5)	3.8 (1.7, 8.7)
Adjusted for in-hospital death (n = 86)	3.7 (1.8, 7.2)	4.1 (2.5, 7.9)	3.3 (1.6, 6.9)
ICU-LOS (ICU admission during hospitalisation) in days, n *=* 327 (MD, IQR)	3.8 (1.7, 8)	4.3 (1.9, 10.1)	3.6 (1.6, 7)
Adjusted for in-hospital death (n = 282)	3.4 (1.6, 6.8)	4.0 (1.9, 8.9) *	3.0 (1.6, 6.0)
In-hospital death (n, %)	82 (4.8)	24 (6)	58 (4.4)
Hospital Acquired Complications:			
Pressure Injury; all (n, %)	16 (0.9)	5 (1.3)	11 (0.8)
Gastrointestinal bleeding; all (n, %)	35 (2.0)	17 (4.3) ^†††^	18 (1.4)
Delirium; all (n, %)	84 (4.9)	31 (7.8) ^††^	53 (4)
Surgical requiring unplanned return to theatre; all (n, %)	74 (4.3)	21 (5.3)	53 (4)
Cardiac; all (n, %)	64 (3.7)	20 (5)	44 (3.3)
Falls resulting in fracture or other intracranial injury; all (n, %)	7 (0.4)	2 (0.5)	5 (0.4)
Healthcare associate infection			
Urinary tract infection (n, %)	55 (3.2)	15 (3.8)	40 (3)
Surgical site infection (n, %)	2 (0.1)	1 (0.3)	1 (0.1)
Blood stream infection (n, %)	19 (1.1)	6 (1.5)	13 (1)
High impact infections (n, %)	69 (4.0)	25 (6.3) ^††^	44 (3.3)
Pneumonia infections (n, %)	109 (6.3)	35 (8.8) ^††^	74 (5.6)
All other healthcare associated infections (n, %)	40 (2.3)	17 (4.3) ^††^	23 (1.7)
Respiratory complications			
Respiratory failure including ARDS (n, %)	41 (2.4)	16 (4) ^††^	25 (1.9)
Aspiration pneumonia (n, %)	34 (2.0)	12 (3) ^†^	22 (1.7)
Pulmonary oedema (n, %)	2 (0.1)	1 (0.3)	1 (0.1)

VCD, vitamin C deficiency; LOS, length of stay; MD, median; IQR, interquartile range, ICU, intensive care unit; ARDS, acute respiratory distress syndrome. Significantly different from the not VCD cohort; * (*p* < 0.05) by Mann–Whitney U test, ^†^ (*p* < 0.10) by Chi square, ^††^ (*p* < 0.05) by Chi square, ^†††^ (*p* < 0.001) by Chi square.

**Table 3:** Univariate and multivariate generalised linear mixed model (GLMM) logistic regression ^1^ analysis to determine demographic and clinical variables associated with VCD (defined as plasma vitamin C level < 11.4 µmol/L) in adult patients admitted into acute care wards (n = 1717).

Demographic and Clinical Predictor Variables	Univariate OR (95% CI)	*p*-Value	Multivariate OR (95% CI)	*p*-Value
Admission age (years)	1.00 (0.99, 1.01)	0.670	--	--
Male gender	1.50 (1.20, 1.88)	<0.001	1.47 (1.17, 1.86)	0.001
Admitted from residential aged care facility	0.32 (0.13, 0.80)	0.016	0.43 (0.17, 1.09)	0.076
Admitted from hospital transfer	1.37 (1.03, 1.81)	0.029	1.17 (0.88, 1.57)	0.278
Respiratory diagnosis	0.50 (0.33, 0.75)	0.001	0.62 (0.40, 0.96)	0.033
Digestive diagnosis	1.73 (1.34, 2.25)	<0.001	1.36 (1.02, 1.82)	0.037
Skin, subcutaneous tissue, and breast diagnosis	0.62 (0.38, 1.00)	0.050	0.75 (0.46, 1.24)	0.263
Protein–energy malnutrition diagnosis	1.72 (1.37, 2.17)	<0.001	1.50 (1.19, 1.91)	<0.001
Surgical procedure	1.65 (1.31, 2.08)	<0.001	1.31 (1.02, 1.68)	0.037
Admission ward-ICU	1.60 (1.02, 2.50)	0.042	1.59 (1.00, 2.52)	0.050

^1^ GLMM logistic regression; variables associated to a degree of *p* < 0.10 at univariate analysis were entered together into a multivariate model, where *p* < 0.05 was considered significant. VCD, vitamin C deficiency; OR, odds ratio; CI, confidence interval; ICU, intensive care unit. Model Summary: The model used a binomial distribution with a logit link function and included a random intercept for acute hospital sites. The Akaike corrected criterion was 8006.142 and the Bayesian criterion was 8011.582. The overall model was significant *F* (9, 1707) = 7.091, *p* < 0.001.

## Data Availability

The data presented in this study may be available on request from the corresponding author due to application and approval from relevant ethics committees.

## Supplementary Materials

The following supporting information can be downloaded at: https://www.mdpi.com/article/10.3390/nu17071131/s1, Table S1. Univariate and multivariate generalised linear mixed (GLMM) model logistic regression analysis to determine if VCD status (defined as plasma vitamin C level < 11.4 µmol/L) is associated with high-impact infection complication in adult patients admitted into acute care wards (n = 1717).
