# Supplementary material for: The Prevalence, Risk Factors, and Clinical Outcomes of Vitamin C Deficiency in Adult Hospitalised Patients: A Retrospective Observational Study
Source: Nutrients. 2025 Mar 25;17(7):1131. doi: 10.3390/nu17071131 (PMC11990434; doi:10.3390/nu17071131)
Supplement: Supplementary file 1 [file nutrients-17-01131-s001.zip › nutrients-3546859-supplementary.pdf]

Supplementary Material:

Table S1. Univariate and multivariate generalised linear mixed (GLMM) model logistic regression[1] analysis to determine if VCD status (defined as plasma vitamin C level <11.4 μmol/l) is associated with high-impact infection complication in adult patients admitted into acute care wards (*n*=1717)

VCD as a predictor for a high-impact infection hospital acquired complication using logistic regression[1]				
Predictor variables	Univariate OR (95% CI)	*p*-value	Multivariate OR (95% CI)	*p*-value
VCD	1.85 (1.12, 3.08)	0.017	1.72 (1.03, 2.87)	0.038
Age at admission (years)	1.01 (1.00, 1.02)	0.113	---	---
Male gender	1.26 (0.78, 2.05)	0.350	---	---
Respiratory diagnosis	0.56 (0.22, 1.42)	0.226	---	---
Infectious diagnosis	0.00 (0.00)	0.958	---	---
Malnutrition diagnosis	1.91 (1.17, 3.14)	0.010	1.80 (1.09, 2.97)	0.021

[1]GLMM logistic regression; variables associated to a degree of p <0.10 at univariate analysis were entered together into a multivariate model, where p <0.05 was considered significant. VCD, vitamin C deficiency; OR, odds ratio; CI, confidence interval. Model Summary: The model used a binomial distribution with a logit link function and included a random intercept for acute hospital sites. The Akaike Corrected criterion was 10,655.939 and the Bayesian criterion was 10,661.383. The overall model was significant F (2, 1714) =5.470, p=0.004.
